# Editorial: Climate Change and Aeroallergens

**DOI:** 10.3389/falgy.2021.794430

**Published:** 2021-12-20

**Authors:** Athanasios Damialis, Matt Smith, Carmen Galán

**Affiliations:** ^1^Department of Ecology, School of Biology, Faculty of Sciences, Aristotle University of Thessaloniki, Thessaloniki, Greece; ^2^School of Science and the Environment, University of Worcester, Worcester, United Kingdom; ^3^Department of Botany, Ecology and Plant Physiology, University of Cordoba, Cordoba, Spain

**Keywords:** aerobiology, climate change, aeroallergens, pollen, fungal spores, temperature, flowering phenology, pollen production

Anthropogenic climate change is likely to affect allergy sufferers by altering the chemical, physical, and biological composition of the atmosphere. For instance, increasing temperatures and changes in precipitation modify plant growth and phenology (e.g., the timing of flowering onset) and, consequently, the start, peak, end and magnitude of the respective pollen seasons as well as the spatial distribution of allergenic plants. Furthermore, increased concentrations of atmospheric carbon dioxide influence plant biomass production, including pollen productivity. Working Group II of the Intergovernmental Panel on Climate Change Fifth Assessment Report (on the assessment of impacts, adaptation, and vulnerability) states that some common allergic diseases, such as asthma, allergic rhinitis, conjunctivitis, and dermatitis, are climate sensitive and climatic change may facilitate the production, release, and dispersal of airborne allergenic pollen and fungal spores. In this Research Topic on Climate Change and Aeroallergens, contributing authors present further evidence on the dramatic effects ongoing global warming has on plants and their reproductive biology and ecology. This is confirmed from studies originating from a variety of climatic zones, namely from temperate to humid subtropical (Levetin; Addison-Smith et al.), to moderate coastal climates to continental (Gehrig and Clot; De Weger et al.) and a range of temperate to continental climates (Menzel et al.), as well as a review from an ecological and botanical perspective (Ziska).

Few studies have focused on long-term changes in allergenic pollen in subtropical climates. Levetin investigated trends in airborne pollen abundances for up to 34 years and for the eight of the most abundant pollen types in the South Central United States, in Tulsa, Oklahoma. Estelle found that maximum air temperature increased over time and so did tree pollen concentrations. This was more pronounced for the pollen of Cupressaceae and *Quercus*. Moreover, pollen seasons started earlier for spring pollinating trees as well as for Poaceae.

Addison-Smith et al. working in subtropical and humid Brisbane, Australia, focused their attention on Poaceae pollen and examined long-term trends from the 1990s to the present day. A remarkable 3-fold increase was found in the seasonal pollen index of Poaceae when comparing the 1990s with the last 5 years. Similar results were also obtained for the frequency of extreme grass pollen days, which occurred more often during the last half decade. In parallel, CO_2_ levels have increased, as to have satellite-derived seasonal vegetation health indices, and daily maximum temperatures.

Gehrig and Clot present a unique dataset from Basel, Switzerland, which is half-a-century-long, since 1969. The authors revealed increases in the temperatures mainly of winter, spring, and summer, of up to almost 4°C, and commensurate advances in the start dates of most pollen seasons in spring (e.g., a week earlier for Poaceae and almost a month earlier for *Taxus*/Cupressaceae. They also found that pollen seasons mostly tended to end earlier, highlighting a shift toward earlier pollen seasons. Apart from the dramatic changes in the seasonality of airborne pollen, the intensity of the pollen season altered too, with almost all tree pollen taxa increasing significantly over time.

de Weger et al. examined a spatially larger area, in the Benelux, and detected long-term trends in airborne pollen abundances over a timespan of up to 44 years. Most examined taxa exhibited an overall increasing trend in the annual pollen integral and peak values, as well as earlier shifts of the pollen seasons. While this was particularly true for the woody taxa, the herbaceous Poaceae and *Artemisia* showed decreases in their annual pollen integral and peak values and had prolonged seasons. These alterations were mostly attributed to specific environmental parameters, like air temperature increases, nevertheless, other trends were more local-specific, thus highlighting the importance of regional impacts such as increasing urbanization.

Based on data from six sites with varying climates in Bavaria, southern Germany, Menzel et al. integrated flowering phenology with airborne pollen measurements over the period 1987–2017. The authors detected significant changes for several taxa. Earlier flowering species showed a more intense advancement of their pollen seasons compared to those flowering later in spring and the same was mostly true for the pollen abundances too. There seemed to be a mismatch between the monitored airborne pollen taxa and the corresponding flowering of local species. In particular, pre-season pollen occurrence was a common incident, with a median of this difference being 17 days and explaining 63% of all cases. Thirty-five percent was confirmed to be long-distance transported pollen. At the alpine station, non-local pollen sources were the rule (mostly originating from outside Bavaria, Germany), with only 13% of the trajectories suggesting local sources of pollen.

All these studies provide further evidence for attributing at least part of the general increase in the global incidence and prevalence of respiratory diseases to climate change. From a more ecological and botanical point of view, it is evident that concurrent increases in CO_2_ and temperature levels boost the plant's capacity of producing pollen. Ziska suggests three significant and interrelated consequences of climate change and other parameters related to global change on respiratory allergies or disease: Firstly, warmer temperatures and longer frost-free growing seasons can influence pollen season length and temporal exposure to airborne aeroallergens; Second, both warmer temperatures and high atmospheric CO_2_ concentrations can increase the amount of pollen produced by the plants; Third, there is evidence from *Ambrosia* and *Quercus* that rising levels of CO_2_ could increase the allergen concentration of the pollen and symptom severity. Given that in real-life conditions it is the entirety of the exposome that defines our diseases and our well-being, additional parameters have to be taken into account to fill potential knowledge gaps. These include, but are not limited to, flower, pollen and allergen production, increasing wind speeds, fungi-host interactions, air quality (e.g., PM, VOCs, O_3_, NO_x_, wildfires) as well as longer-term habitat shifts and loss, vernalization, and masting.

## Author Contributions

AD wrote the original draft. AD, MS, and CG reviewed and approved the final draft. All authors contributed to the article and approved the submitted version.

## Conflict of Interest

The authors declare that the research was conducted in the absence of any commercial or financial relationships that could be construed as a potential conflict of interest.

## Publisher's Note

All claims expressed in this article are solely those of the authors and do not necessarily represent those of their affiliated organizations, or those of the publisher, the editors and the reviewers. Any product that may be evaluated in this article, or claim that may be made by its manufacturer, is not guaranteed or endorsed by the publisher.

